# Association of Neuroblastoma (NB) SH-SY5Y Cells with Antibodies of Parasitic Origin (Anti-*Acanthamoeba* and Anti-*Toxocara canis*)

**DOI:** 10.3390/ijms252413577

**Published:** 2024-12-19

**Authors:** Víctor Alberto Maravelez Acosta, Maria de Lourdes Caballero Garcia, Genaro Patiño López, María del Pilar Crisóstomo Vázquez, Luz Ofelia Franco Sandoval, Leticia Eligio García

**Affiliations:** 1 Laboratorio de Investigación en Parasitología, Hospital Infantil de México Federico Gómez (HIMFG), Dr. Márquez 162. Col Doctores, Cuauhtémoc, México City 06720, Mexico; maravelez@hotmail.com (V.A.M.A.);; 2 Unidad de Investigación en Inmunología y Proteomica, Hospital Infantil de México Federico Gómez (HIMFG), Dr. Márquez 162. Col Doctores, Cuauhtémoc, México City 06720, Mexico

**Keywords:** neuroblastoma, *Acanthamoeba*, *Toxocara canis*, antibodies and antitumor effect

## Abstract

It is little known that *Acanthamoeba* trophozoites and *Toxocara canis* eggs can reduce tumors in vitro and animal models. Although this has been known for many years, the mechanism that induces the antitumor effect in these parasites is still not known. We employed Western blot (WB) and immunofluorescence (IFC) by confocal microscopy to explore the potential protein binding between neuroblastoma (NB) SH-SY5Y cells and anti-*Acanthamoeba* and anti-*Toxocara canis* antibodies. Using WB, we detected two fragments of 70 kDa and 60 kDa recognized by the anti-*Acanthamoeba* antibodies, and two fragments of 115 kDa and 70 kDa recognized by the anti-*Toxocara canis* antibodies. In both cases, the IFC results were positive in the cell membrane of the SH-SY5Y cells. Our findings suggest a potential overlap of similar molecules between these parasites and tumor cells, which may contribute to tumor elimination. Investigating the relationship between anti-*Acanthamoeba* and anti-*Toxocara canis* antibodies in neoplastic cells could provide evidence for the future use of these anti-parasitic antibodies in targeting NB or other cancers.

## 1. Introduction

Neuroblastoma (NB), the most common extracranial solid tumor in children under the age of 5, was described as early as the 19th century, and its complexity has continued to intrigue researchers, as well as medical and surgical specialists. At one end of the phenotypic spectrum, neuroblastoma is self-limiting with minimal to no intervention required, while on the opposite end exists the challenge of refractory disease despite aggressive management and toxic systemic treatments [1]. Immunotherapy has faced challenges in pediatric patients, and although the etiology of neuroblastoma is multifactorial, a significant portion of the lack of response is thought to be attributed to the tumor microenvironment [2]. A prominent immunotherapeutic strategy in NB involves using antibodies against the tumor-associated disialoganglioside GD2. This strategy is used due to the fact that the Fc portion of the anti-GD2 antibodies, which binds to neuroblastoma tumor antigens, is recognized by natural killer (NK) cells, through antibody-dependent cell-mediated cytotoxicity (ADCC) [3]. It is possible to combine immunotherapy with standard chemotherapy or other immunomodulatory agents to enhance the therapeutic effect. Additionally, research is being conducted to prolong the efficacy and potency of therapies aimed at improving the expansion and activation of NK cells [4]. Despite recent advances in the management and therapeutics of cancer, the treatment of the disease is limited by its excessive cost and severe side effects. In this scenario, there is an unmet need to identify novel treatment alternatives for this dreaded disease [5].

Several parasites have shown the ability to slow certain types of cancer growth [6]. Table 1 presents the parasites that have been most extensively studied for their antitumor effects.

A little-known fact is that *Acanthamoeba* and *Toxocara canis* have been associated with possible anticancer activity.

*Acanthamoeba* is a unicellular protozoan and has an evolutionary history that spans at least a billion years [49]; its pathological significance was acknowledged in the 1960s and 1970s, and it was recognized as the etiological agent of amoebic granulomatous encephalitis and keratitis [50]. *Acanthamoeba* is widely distributed in nature, existing in the form of free-living organisms or parasites, and is frequently associated with biofilms in various environments. Since 1986, *Acanthamoeba* has emerged as a global public health concern due to the use of contact lenses, although they have historically caused disease in immunosuppressed individuals [51]. *Acanthamoeba* can interact with various microorganisms, including bacteria, fungi and viruses [52]. As a result, *Acanthamoeba* can act as a predator, a vehicle for transmission, or an incubator in natural environments [53]. *Acanthamoeba* feeds on bacteria by absorbing them through phagocytosis and subsequently lysing them in phagolysosomes. Additionally, *Acanthamoeba* can function as a ‘Trojan horse’, serving as a vehicle for microbial transmission in the environment. In other words, *Acanthamoeba* can incubate microorganisms that are pathogenic to humans. These microorganisms utilize the parasite’s defense mechanisms to evade the immune system and treatment while reproducing freely within it. Consequently, *Acanthamoeba* serves as a ‘genetic melting pot’, promoting gene exchange and the adaptation of microorganisms, which in turn enhances their pathogenicity [52]. *Acanthamoeba castellanii* trophozoites demonstrated a strong chemotactic response toward human melanoma (OCM-1) and murine melanoma (D5.1G4) cells. This response was observed by injecting either live parasites or cell-free parasite lysates into a melanoma animal model, which led to a reduction in tumor mass between 53% and 83% [54]. Murine NB cells (NB41A3) were exposed to a parasite extract of *Acanthamoeba castellanii*. The results indicated that over 70% of NB41A3 DNA was fragmented, and several morphological features of apoptosis were observed, including cell shrinkage, vesicle formation on the cell membrane, the formation of apoptotic bodies, and nuclear condensation. The results suggest that at least one species of pathogenic free-living amoeba can lyse tumor cells by a process that culminates in apoptosis [55]. The cytotoxic effect of five isolates of *Acanthamoeba* (trophozoítes) on human cervix cancer HeLa cells culture was investigated. The HeLa cells showed a sequence of morphological features of apoptosis and 50% of the tumor cells underwent cytolysis. These five strains of *Acanthamoeba* exhibit cytotoxic effects of varying degrees on HeLa cells [56]. Signs of apoptosis were observed when NB cells were exposed to an isolate of *Acanthamoeba* known as AS. Notable features included cell contraction, nuclear condensation, and DNA fragmentation. Apoptosis was determined to be mediated by the involvement of caspase enzymes and the pro-apoptotic and anti-apoptotic mitochondrial proteins Bax and Bcl-2 [57]. The effect of cell-free supernatants that was obtained from four axenic cultures of *Acanthamoeba* (trophozoítes) was investigated on three cancer cell lines (human prostate cancer, rat prostate cancer and breast cancer). All analyzed samples demonstrated an inhibitory effect on prostate cancer cells; however, this effect was not observed in breast cancer cells [58].

*Toxocara canis* infection (toxocariasis) is a cosmopolitan, zoonotic and neglected disease [59,60]. Toxocariasis is an infection caused in canines, felines, humans and other vertebrates by species of the genus *Toxocara*, such as *Toxocara canis* and *Toxocara cati* [61]. The definitive hosts are domestic or wild canids (such as dogs), and due to perinatal transmission, it also affects puppies under three months of age [62,63]. The primary mode of infection acquisition is through the ingestion of embryonated eggs, affecting both definitive hosts (dogs and cats) and paratenic hosts, including humans [61]. *Toxocara canis* has been shown to have both antitumor and protumor growth properties [9,10,11,12,13,14,15,16,17,18,19,20,21,22,23,24,25,26,27,28,29,30,31,32,33,34,35,36,37,38,39,40,41,42,43,44,45,46,47,48,49,50,51,52,53,54,55,56,57,58,59,60,61,62,63,64,65,66]. *Toxocara canis* egg antigens induced the inhibition of tumor growth in the fibrosarcoma mouse model; the mean tumor area in *Toxocara canis*-injected mice over four different days was 24.5 mm^2^ compared to the control group (alum treated) which was 155 mm^2^ [9]. A peptide sequence was synthesized from an excretory–secretory antigen of *Toxocara canis* (TOXCA Troponin T protein) and the possible anticancer properties and their effect on gastrointestinal and liver cancer cell proliferation-related genes in laboratory conditions were evaluated. The peptide, when administered at high concentrations, promotes cancer cell mortality and alters gene expression related to apoptosis, metastasis and angiogenesis [24]. Due to their ability to alter the host’s immune response and their widespread presence in the environment, helminths have been found to promote tumor growth. This has been demonstrated in studies involving *Schistosoma haematobium*, *Clonorchis sinensis* and *Opisthorchis viverrine* [65,66]. *Toxocara canis* exhibits immunomodulatory properties that enable it to infect humans and other hosts [67]. By modulating the immune response, *Toxocara canis* can influence tumor growth in mice, leading to the development of larger tumors; in other words, *Toxocara canis* modulates the immune microenvironment of the tumor [68]. The intratumoral injection of *Toxocara canis* excretory/secretory antigens (EST) promotes lung metastasis through the modulation of the tumor immune microenvironment in mice. ESTs were injected intratumorally into mice with lung cancer, resulting in no significant changes in tumor size or weight. However, there was a marked increase in microvasculature and the development of both micro- and macro-metastases in the lungs, along with elevated levels of VEGF [69]. *Toxocara canis* increases the potential of breast cancer by reducing the expression of P53, a tumor suppressor protein, and increases the Ki-67 protein; this means that cancer cells divide rapidly [70]. Evidence suggests that *Toxocara canis* has an antitumor effect in its egg form and a protumor effect during infection in its larval form.

*Acanthamoeba* trophozoite and *Toxocara canis* egg antigens induce apoptosis in neoplastic cells, but which parasite antigens interfere with tumor growth has not been investigated. For this reason, we focused on first identifying whether there is a recognition of anti-*Acanthamoeba* and anti-*Toxocara canis* antibodies on the membranes of SH-5S5Y and then detected possible proteins that have unexpected cross-linking between anti-*Acanthamoeba*/anti-*Toxocara canis* antibodies in the antigens of SH-5S5Y cells.

## 2. Results

### 2.1. Recognition of Anti-Acanthamoeba and Anti-Toxocara canis Antibodies on the Total Proteins of SH-5S5Y

**Figure 1:**
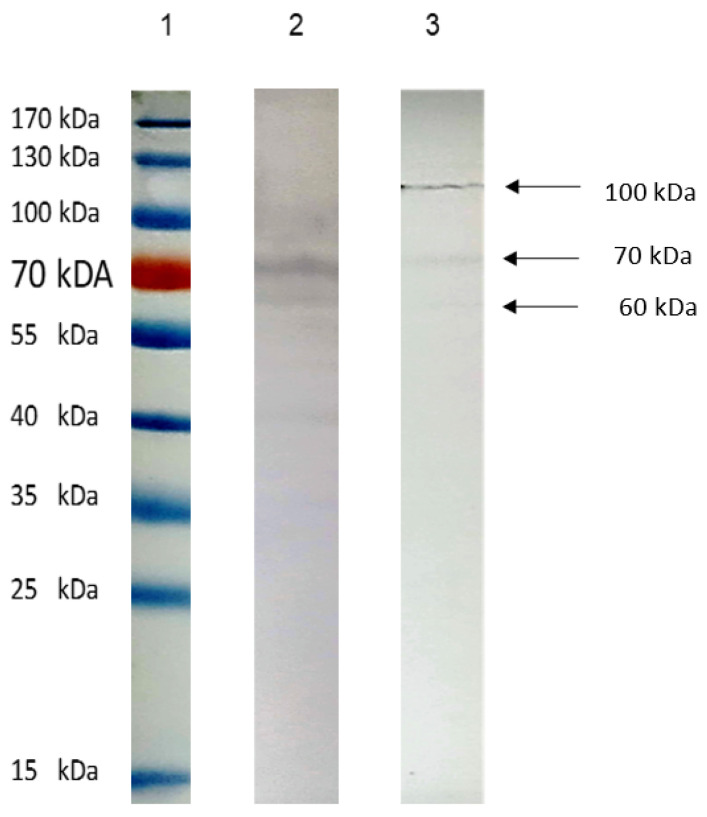
Protein profile and recognition of SH-5S5Y with anti-*Acanthamoeba* and anti-*Toxocara canis* antibodies by WB. Shown: (1) molecular weight and protein extract of SH-5S5Y (1000 μg). Preparation was separated by SDS_PAGE 12% and electro-transferred to nitrocellulose membranes. Reactivity was carried out with (2) anti-*Acanthamoeba* and (3) anti-*Toxocara canis*. Anti-rabbit IgG coupled to peroxidase 1:10,000 was used for immunostaining and rebelled with 4-chloro-naphthol.

To identify SH-5S5Y proteins recognized by anti-*Acanthamoeba* and anti-*Toxocara canis* antibodies, we performed WB analysis using total protein extracts of SH-5S5Y against a panel of anti-*Acanthamoeba* and anti-*Toxocara canis* antibodies, both at a concentration of 8.8 μg/μL and at a dilution of 1:100. Prominent fragments were consistently detected (Figure 1); we found two fragments of 70 Kdal and 60 Kdal recognized by the anti-*Acanthamoeba* antibodies and two fragments of 115 Kdal and 70 Kdal recognized by the anti-*Toxocara canis* antibodies. These findings suggest that anti-*Acanthamoeba* and anti-*Toxocara canis* antibodies may effectively target the specific antigens that are present within SH-5S5Y.

### 2.2. Recognition of Anti-Acanthamoeba and Anti-Toxocara canis Antibodies on the Membrane of SH-5S5Y

**Figure 2:**
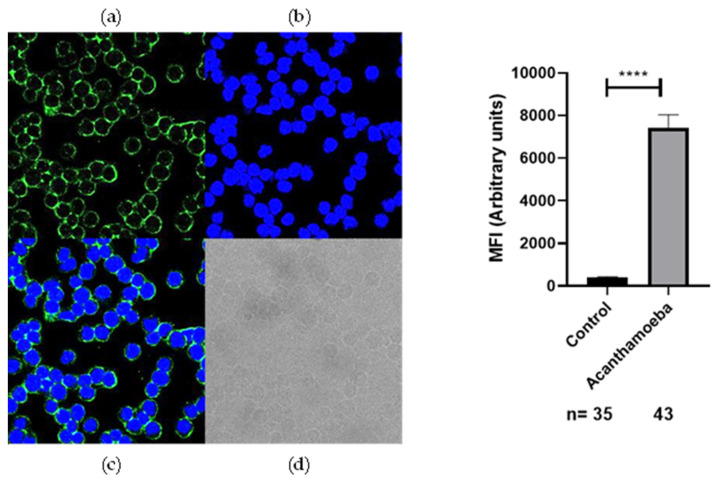
IFC by confocal microscopy. (**Left**), recognition of membrane antigens of SH-5S5Y with specific anti-*Acanthamoeba* antibodies. SH-SY5Y cells were incubated with anti-*Acanthamoeba* antibodies (8.8 μg/µL, diluted 1:300), Alexa 488-conjugated secondary antibodies (1:300) and DAPI for nuclear staining. The images shown include the following: (**a**) Alexa-488 staining, (**b**) DAPI nuclear staining, (**c**) Merge, Alexa-488 and DAPI, (**d**) black and white representation. All images were captured using a 63X objective lens with 2X digital magnification. (**Right**), fluorescence intensity quantification for anti-*Acanthamoeba* antibodies alongside its respective control (rabbit preimmune IgG). *p* < 0.0001 (****).

**Figure 3:**
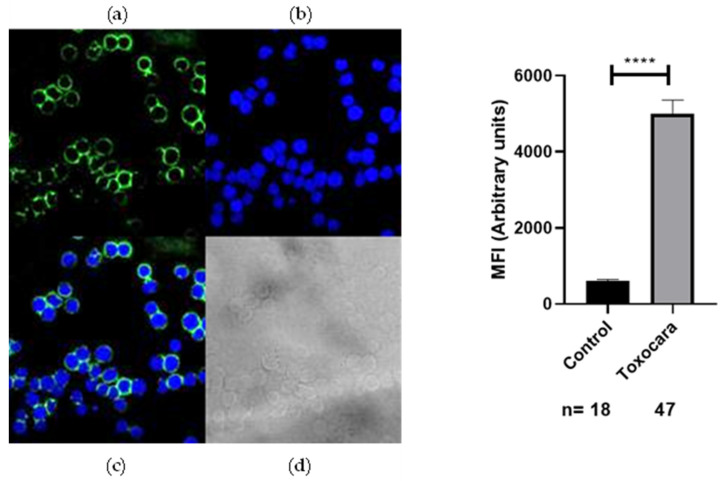
IFC by confocal microscopy. (**Left**), recognition of membrane antigens of SH-5S5Y with specific anti-*Toxocara canis* antibodies. SH-SY5Y cells were incubated with anti-*Toxocara canis* antibodies (8.8 μg/µL, diluted 1:300), Alexa 488-conjugated secondary antibodies (1:300) and DAPI for nuclear staining. The images shown include the following: (**a**) Alexa-488 staining, (**b**) DAPI nuclear staining, (**c**) Merge, Alexa-488 and DAPI, (**d**) black and white representation. All images were captured using a 63X objective lens with 2X digital magnification. (**Right**), fluorescence intensity quantification for anti-*Toxocara canis* antibodies alongside its respective control (rabbit preimmune IgG). *p* < 0.0001 (****).

To investigate the recognition of anti-*Acanthamoeba* and anti-*Toxocara canis*-specific antibodies by the membrane protein SH-5S5Y, IFC analysis was performed. We evaluated non-permeabilized SH-5S5Y. Anti-*Acanthamoeba* and anti-*Toxocara canis* antibodies (8.8 μg/µL) were diluted (1:300) and incubated with SH-5S5Y-expressing cells. The binding of these antibodies was detected using Alexa Fluor 488-conjugated anti-rabbit IgG antibodies (1:300). The results indicated that SH-5S5Y-expressing cells displayed positive staining for anti-*Acanthamoeba* (Figure 2) and anti-*Toxocara canis* (Figure 3) in the membrane of the cells. These findings suggest that a substantial proportion of SH-5S5Y molecules on the cell surface are recognized by anti-*Acanthamoeba*/anti-*Toxocara canis*-specific antibodies. The fluorescence intensity quantification for anti-*Acanthamoeba* and anti-*Toxocara canis* antibodies was analyzed alongside their respective control (rabbit preimmune IgG). The statistical analysis shows a *p*-value of <0.0001 (****) for both antibodies, indicating an extremely statistically significant result. Figure 2 and Figure 3 display the graphs of fluorescence intensity quantification for each antibody alongside its respective control.

## 3. Discussion

In studies conducted with *Acanthamoeba*, it has been demonstrated that the antigens of the parasite, when injected into a murine melanoma model, suppress tumor cells [54]. Similarly, in *Toxocara canis*, antigens have been shown to suppress tumor cells in a murine fibrosarcoma model [9]. The antitumoral effect of *Acanthamoeba* and *Toxocara canis* has ceased to be a subject of study, and the mechanisms involved in tumor suppression have not yet been defined. The role of anti-*Acanthamoeba* and anti-*Toxocara canis* antibodies in this antitumoral effect has not been studied. This is the first study to analyze the potential of antibodies induced by *Acantamoeba* trophozoites and *Toxocara canis* eggs antigens on cancer cells. In this study, neuroblastoma cells from the SH-5S5Y cell line were used.

To better understand the possible usefulness of using anti *Acantamoeba* and anti *Toxocara canis* antibodies to recognize the antigens of SH-5S5Y, we performed WB, where we used the total proteins of SH-5S5Y vs. the anti-*Acantamoeba* and anti-*Toxocara canis* antibodies. The results demonstrate that there is a cross-linking between anti-*Acantamoeba* and anti-*Toxocara canis* antibodies with SH-5S5Y antigens; see Figure 1. We identified two bands of approximately 70 Kdal and 60 Kdal that cross-link with the anti-*Acantamoeba* antibodies, and two bands of approximately 115 Kdal and 70 Kdal that cross-link with the anti-*Toxocara canis* antibodies. Each band contains a mixture of proteins, where one or more may contribute to the antitumor effect. Another strategy involved analyzing whether the anti-*Acanthamoeba* and anti-*Toxocara canis* antibodies could specifically recognize antigens on the SH-5S5Y membrane; therefore, we conducted IFC by confocal microscopy. We also found that the membrane antigens of SH-5S5Y specifically recognize the anti-*Acantamoeba* antibodies Figure 2 and the anti-*Toxocara canis* antibodies Figure 3. The results of the IFC were consistent for both antibodies, showing A clear recognition of anti-*Acantamoeba* and anti-*Toxocara canis* antibodies binding to the antigens present in the membrane of SH-5S5Y. The statistical analysis of fluorescence intensity quantification for the anti-*Acanthamoeba* and anti-*Toxocara canis* antibodies shows a *p*-value of <0.0001 (****) for both antibodies, indicating an extremely statistically significant result. Figure 2 and Figure 3 present the fluorescence intensity quantification graphs for each antibody alongside its respective control. The recognition of SH-5S5Y membrane antigens by anti-*Acantamoeba* and anti-*Toxocara* antibodies suggests that these antibodies may be involved in cellular cytotoxicity mechanisms against cancer cells, potentially leading to a reduction in tumor mass, as demonstrated in studies utilizing murine models of melanoma and fibrosarcoma [54,58].

The antitumor effect of *Acantamoeba* on NB cells has been previously studied, revealing that (i) antigens from lysate trophozoites induce apoptosis in cultured NB cells [55] and (ii) in co-culture experiments involving trophozoites and NB cells, AS trophozoites (T4) attacked the NB cell monolayers immediately upon addition, penetrated the monolayers, and induced apoptosis in NB cells via the Bax pathway [57]. Additionally, we have demonstrated that anti-*Acanthamoeba* antibodies exhibit cross-linking with both total antigens and the antigens present in the SH-5S5Y membrane, highlighting an intriguing direction for further study. In a previous study, *Acanthamoeba* was examined in vivo and it was revealed that the injection of trophozoites lysates into progressively growing subcutaneous melanomas resulted in 83% and 53% reductions in the tumor masses [54]; in other words, anti-*Acantamoeba* antibodies were generated and contributed to tumor reduction through the cross-recognition of antigens present in cancer cells.

There is evidence that some helminths are inductors of different types of tumors [71]. *Toxocara canis* chronic infection in BALB/c mice results in a type 2 response with an incipient regulatory response [71], and chronic infection with *Toxocara canis* in BALB/c mice with breast cancer accelerates tumor growth and is associated with alterations in the tumor microenvironment. The higher proportions of immune cells found in the tumor microenvironment comprised F4/80+ macrophages and CD19+ B cells, which could contribute to tumor enlargement and reduce the proportions of CD8+ lymphocytes. Furthermore, it was found that the IL-4 and VEGF levels were elevated, and a microenvironment characterized by higher levels of IL-10 was observed. This study demonstrated that *Toxocara canis* infection promotes a protumor effect through the modulation of the tumor immune microenvironment [69]. Additionally, the intratumoral injection of excretion/secretion antigens from *Toxocara canis* (EST) in lung cancer models induces a protumoral effect, without significant changes in tumor size or weight; it was noted that lung tumors exhibited increased micro- and macro-metastasis, as well as enhanced microvasculature. In contrast, EST did not alter the proportions of immune cells in the tumor, spleen, or peripheral lymph nodes. Therefore, the effect of EST was to promote lung metastasis by modulating the tumor’s immune microenvironment [64]. Therefore, *Toxocara canis* infection in a murine model of breast cancer, as well as the intratumoral injection of EST in a lung cancer model, alters the tumor microenvironment, thereby promoting a protumoral effect. Helminths have been recognized as inducers or promoters of cancer due to their ability to regulate the host’s immune response [69,70]. In contrast, we utilize a total extract of *Toxocara canis* antigens derived from larvae eggs to generate antibodies. ESTs were not included in our experiments, as we did not collect them; however, we do not rule out the possibility that small amounts could be present within the parasite before secretion. Nonetheless, larval *Toxocara canis* eggs have never been shown to provide the conditions necessary to stimulate the production of EST. We found that anti-*Toxocara canis* antibodies recognized SH-5S5Y antigens Figure 1 and Figure 3, suggesting that these antibodies may be associated with the antitumor effect of *Toxocara canis* due to the similarity between the proteins of the parasite and those of SH-5S5Y. *Toxocara canis* egg antigens inhibited tumor growth in a fibrosarcoma mouse model, likely because the immune responses elicited by parasite antigens may exert non-specific effects on tumor cells [9]. By comparing our results with those of previous studies, we can deduce that the antitumor effect of *Toxocara canis* may be attributed to the ability of anti-*Toxocara canis* antibodies to recognize antigens on the SH-5S5Y membrane and potentially participate in therapeutic mechanisms such as antibody-dependent cellular cytotoxicity (ADCC) or complement-dependent cytotoxicity (CDC) against cancer cells. In the case of other parasites with antitumor effects, such as *Trypanosoma cruzi*, total antigens, antibodies and induced infections in mice have demonstrated antitumor activity [35,72,73,74]. We did not observe this effect with *Toxocara canis*. Although it has been established that helminths are associated with cancer induction [66], we find a duality: (i) the in vivo infections in mice and the EST of the parasite modify the tumor microenvironment and promote tumor growth [69]; on the other hand, (ii) antigens of the parasite that are absent from ESTs could induce an antitumor effect in vivo tests [9]. Our results indicate that the antitumor effect of *Toxocara canis* could be attributed to the cross-linking between anti-*Toxocara canis* antibodies and cancer cell antigens. This interaction could initiate mechanisms involving antibodies, such as ADCC and CDC. Our results also suggest the possibility that helminth lysates may exert an antitumor effect, contrasting with the protumor effects associated with the helminth infections of EST. However, this remains a hypothesis that requires thorough investigation. The involvement of *Trypanosoma cruzi* in the antitumor effect can elicit both humoral and cellular responses [36]. This suggests that a similar response could occur with *Acantamoeba* and *Toxocara canis* antigens.

The cross-linking between anti-*Acantamoeba* and anti-*Toxocara canis* antibodies and SH-5S5Y cell antigens could be responsible for the tumor cell suppressor effect, since there are similar antigens with SH-5S5Y. In future research, it will be essential to determine the cytotoxic potential (ADCC and CDC) of anti-*Acantamoeba* and anti-*Toxocara canis* antibodies on NB cells and other cancer types. Additionally, identifying the specific proteins involved in tumor suppression is essential, as this knowledge could help advance the development of future cancer therapies based on parasitic-origin molecules. The antitumor effects of *Acanthamoeba* and *Toxocara canis* require further investigation. This work brings us closer to establishing that antibodies induced by *Acanthamoeba* and *Toxocara canis* antigens play a role in the antitumor effect.

## 4. Materials and Methods

### 4.1. Cell Line of SH-SY5Y and Obtaining the Antigen

The NB cell lines SH-SY5Y were obtained from ATCC (Manassas, VA, USA). The cell line was cultured in DMEM media (Gibco, Gran Island, NY, USA) supplemented with 10% fetal calf serum (Gibco, NY, USA) and 1X Antibiotic-Antimycotic (Gibco, Gran Island, NY, USA). These were counted in a Neubauer chamber and assessed for viability with trypan blue.

### 4.2. Acanthamoeba Trophozoites Culture and Obtaining the Antigen

*Acanthamoeba* trophozoites were obtained from viable cysts stored in Petri dishes with non-nutritive agar at 4 °C. For excystment, a 1 × 0.5 cm gel sample was taken and sown in Petri dishes with non-nutritive agar (NaCL 0.06 g, magnesium sulfate 0.002, anhydrous dibasic sodium phosphate 0.007 g, bacteriological agar 7.5 g and monobasic potassium phosphate 0.068 g) together with 100 uL of culture (5% meat peptone) saturated with inactivated *E. coli* for 1 h at 56 °C and incubated at 37 °C for 96 h for excystment. The trophozoites were expanded in PBSMG medium (peptone biotryptase 8.3 g, dextrose 1.3 g, anhydrous dibasic sodium phosphate 0.75 g, monobasic potassium phosphate 0.45 g and 10% FBS) for 7 days at 28 °C. To obtain the antigen, the *Acanthamoeba* trophozoites were harvested and washed. The trophozoites were counted in a Neubauer chamber and the viability was assessed with trypan blue. To extract the antigen subsequently, the cell pellet was resuspended in cold protein lysis buffer (1% Nonidet P-40, 150 mM NaCl, 10 mM Tris-HCl pH 7.6, 10 mM PMSF, 2 mM EDTA, Protinin A 1 µg/mL, Pestatin A 1 µg/mL y Leupeptin A 1 µg/mL) and lysed by freezing for 30 min. The total antigen concentration obtained was determined on a UV-Visible spectrophotometer at 280 nm Epoch (Biotec Instruments, Winooski, VT, USA) [72].

### 4.3. Toxocara canis Eggs Culture and Obtaining the Antigen

To obtain *Toxocara canis* eggs, the small intestine of a puppy sacrificed by the Canine Control Center of CDMX, Mexico, was recovered. The ends of the intestine were tied together for transport. In the laboratory, the intestine was cut into fragments of approximately 3 cm; subsequently, each fragment was cut longitudinally. When *Toxocara canis* was identified, it was placed in culture flasks with 1X PBS. To obtain embryonic eggs, 2 females and 2 males were placed in a culture box with warm 1X PBS and incubated at 37 °C for 24 h. The next day, all of the PBS were collected and centrifuged at 2500 rpm for 5 min and the larvae eggs were recovered. EST were not considered in this study. The eggs were counted in a Neubauer chamber. Subsequently, to extract the antigen, the cell pellet was resuspended in cold protein lysis buffer (1% Nonidet P-40, 150 mM NaCl, 10 mM Tris-HCl pH 7.6, 10 mM PMSF, 2 mM EDTA, Protinin A 1 µg/mL, Pestatin A 1 µg/mL y Leupeptin A 1 µg/mL) and mechanically lysed in a mortar under freezing conditions for 30 min using liquid nitrogen. The total antigen concentration obtained was determined on a UV-Visible spectrophotometer at 280 nm Epoch (Biotec Instruments, Winooski, VT, USA) [72].

### 4.4. Generation of Anti-Acanthamoeba and Anti-Toxocara canis In Vivo with New Zeeland Rabbits

Six-week-old rabbits were obtained from of the animal facility of the Children’s Hospital de Mexico Federico Gomez (Mexico City, Mexico). The animal study protocol was approved by the Ethics Committee of the Children’s Hospital of Mexico Federico Gomez (HIM-2014-035) and follows the guidelines for animal experiments in Mexico. Antibody induction was conducted separately using proteins derived from *Acanthamoeba* trophozoites and *Toxocara canis* eggs as immunogens. At time 0, the rabbits were injected with 2 mg of antigen dissolved in 150 µL physiological solution saline via an intradermal route and were emulsified with 150 µL Freud’s complete adjuvant (Sigma, St. Louis, MO, USA); on day 15, 2 mg of antigen dissolved in 150 µL physiological solution saline was injected via an intradermal route and emulsified with 150 µL Freud’s complete adjuvant (Sigma, St. Louis, MO, USA). On day 30, the rabbits were injected intravenously with 0.25 mg antigen dissolved in 150 µL physiological solution saline, on day 31, they were injected intravenously with 0.50 mg antigen dissolved in 150 µL physiological solution saline and on day 32, they were injected intravenously with0.50 mg antigen dissolved in 150 µL physiological solution saline. The total collection of the serum was on day 39 [72].

### 4.5. Purification of Anti-Acanthamoeba and Anti-Toxocara canis Antibodies by Affinity Chromatography with Protein A/G

IgG purification followed the manufacturer’s protocol from Thermo Scientific, using affinity chromatography with protein A/G using Spin Columns (Thermo Scientific, Rockford, IL, USA). The concentrations of purified antibodies were quantified by measuring the absorbance of each fraction at 280 nm in an Epoch (Biotec Instruments, Winooski, VT, USA) UV/Vis spectrophotometer [72].

### 4.6. Identification of SH-SY5Y Proteins Recognized by Anti-Acanthamoeba and Anti-Toxocara canis Antibodies by Western Blotting

To identify the proteins of the SH-SY5Y that are recognized by anti-*Acanthamoeba* and anti-*Toxocara canis* antibodies, Western blotting was performed. The separating gel was prepared with 12% acrylamide–bisacrylamide (Sigma, Amsterdam, The Netherlands) and the concentrator gel with 4% acrylamide–bisacrylamide. The samples were prepared 1:5 with sample buffer under reducing conditions. The voltage was set to 200 V (constant) for 50 min. The electrophoresis gel was placed on a 0.2 µM nitrocellulose membrane (Amersham Biosciences, Region, UK) using the Trans-Blot-Transfer (Bio-Rad, Hercules, CA, USA) chamber cassette; the voltage was conditioned at 100 V for 2 h. To verify the proper transfer of proteins to the nitrocellulose membrane, the membrane was stained with 0.2% Ponceau red and washed with water until the dye was completely removed. The dilution ratio of the anti-*Acanthamoeba* and anti-*Toxocara canis* antibodies was 1:100 overnight, both starting at a concentration of 8.8 μg/μL. The rabbit anti-IgG antibody coupled to horseradish peroxidase (Sigma, Jerusalem, Israel) was at a 1:5000 dilution for 2 h. The blot was developed with 4-CN (Sigma, USA) and H_2_O_2_ (ProdQuimMonterrey, Monterrey, Mexico) [72].

### 4.7. Determination of Anti-Acantamoeba and Anti-Toxocara canis Antibodies Recognition with Neuroblastom Cells by Immunofluorescence in Confocal Microscopy

For evaluating the recognition of anti-*Acantamoeba* and anti-*Toxocara canis* antibodies by IFC analyses, the line cell SH-SY5Y was used. The used dilution of the anti-*Acantamoeba* and anti-*Toxocara canis* antibodies was 1:300 for 1 h, both starting at a concentration of 8.8 μg/μL. For reactivity, specific anti-rabbit IgG antibodies conjugated to Alexa-Fluor 488 (Invitrogen, Lane County, OR, USA) were used and diluted 1:300 for 1 h. Core staining was carried out with 20 µL of mounting medium with 4′,6-diamidino-2-phenylindole diluent (DAPI) (Vector Laboratories, Newark, CA, USA). Images were captured using a TCS-5P8X (Leica, Microsystems, Wetzlar, Germany) confocal microscope and analyzed with Fiji software version 1.54k (https://imagej.net/software/fiji/downloads (accessed on 22 October 2024)) and included the analysis of fluorescence intensity quantification.

## Figures and Tables

**Table 1:** The parasites that have been most studied for their antitumor effects.

Parasite	Antitumor Effect
*Toxoplasma gondii*	Breast cancer, prostate cancer DU-145 cells and lung cancer cells A549 [7], murine sarcoma 180 cells [8], fibrosarcoma WEHI-164 cells [9], human melanoma B16-F10 cells [10], mouse melanoma [11], ovarian cancer A2780 and resistant A2780-CP cells [12], human glioma U373MG and U87MG cells [13], medulloblastoma Shh-subtype cells [14], colorectal carcinoma CT26 cells [15], human gastric cancer BGC-823 cells [16], hepatocellular carcinoma H7402 cells [17] and human chronic myeloid leukemia K562 cells [18].
*Trichinella spiralis*	Murin sarcoma cells 180, hepatoma H22 and H7402 cells murine forestomach carcinoma MFC cells and human chronic myeloid leukemia K562 cells [19], human osteosarcoma MG-63 cells [20], melanoma B16-F10 cells [21], human cervical carcinoma HeLa and T24 cells, human transitional cell bladder carcinoma, IV grade [22], HCT-8 human colorectal carcinoma [23], lung cancer H446 SCLC cells [24] and breast cancer [25].
*Plasmodium yoelii*	Human melanoma B16-F10 cells [26], mouse glioma GL261 cells and lung cancer LLC cells [27], colon cancer cells and murin melanoma B16-F10 cells [28]. Murine Lewis lung cancer cells [29], murine triple-negative breast cancer [30], hepatoma cells [31] and murine WEHI-3 leukemia cells [32].
*Trypanosoma cruzi*	Breast cancer cell [33], murine melanoma B16-F10 cells [34], human neuroblastoma SH.5S5Y cells and human leukemia SUPB15 cells [35], colon and mammary rat cancer cells and human colon cancer cells [36] and murine lung cancer [37].
*Echinococcus granulosus*	Human melanoma cancer A375 cells [38], breast cancer MDA-MB-231, MCF-7 and T47D cells [39], mouse colon cancer C26 cells [40], pancreas cancer induced in a rat [41], lung cancer HCL-H209/Anl cells [42], murine fibrosarcoma WEHI-164 cells [43] and cronic myeloid leukemia K562 cells [44].
*Trichomonas vaginalis*	Human cervical carcinoma HeLa cells [45], prostate cancer PC-3 and DU145 cells [46] and human lung alveolar basal carcinoma epithelial A549 cells [47].
*Schistosoma mansoni*	Murine sarcoma 180 cells [48], murine fibrosarcoma WEHI-164 cells [43] and DMH-induced colon carcinogenesis [20].

## Data Availability

Data are contained within the article.
